# Potential Small Molecules for Therapy of Lupus Nephritis Based on Genetic Effect and Immune Infiltration

**DOI:** 10.1155/2022/2259164

**Published:** 2022-04-23

**Authors:** Jianbo Qing, Wenzhu Song, Lingling Tian, Sonia Biju Samuel, Yafeng Li

**Affiliations:** ^1^The Fifth Clinical Medical College of Shanxi Medical University, Taiyuan, Shanxi 030001, China; ^2^School of Public Health, Shanxi Medical University, Taiyuan, Shanxi 030001, China; ^3^Shanxi University of Traditional Chinese Medicine, Taiyuan, Shanxi 030000, China; ^4^Department of Medicine, Albany Medical Center. 43 New Scotland Ave, Albany, New York 12208, USA; ^5^Department of Nephrology, Shanxi Provincial People's Hospital (Fifth Hospital) of Shanxi Medical University, Taiyuan, Shanxi 030012, China; ^6^Core Laboratory, Shanxi Provincial People's Hospital (Fifth Hospital) of Shanxi Medical University, Taiyuan, Shanxi 030012, China; ^7^Shanxi Provincial Key Laboratory of Kidney Disease, Shanxi Provincial People's Hospital (Fifth Hospital) of Shanxi Medical University, Taiyuan, Shanxi 030012, China; ^8^Academy of Microbial Ecology, Shanxi Medical University, Taiyuan, Shanxi 030000, China

## Abstract

Lupus nephritis (LN) is the most common and significant complication of systemic lupus erythematosus (SLE) due to its poor prognosis and mortality rates in SLE patients. There is a critical need for new drugs as the pathogenesis of LN remains to be elucidated and immunosuppressive therapy comes with many deficiencies. In this study, 23 hub genes (IFI6, PLSCR1, XAF1, IFI16, IFI44, MX1, IFI44L, IFIT3, IFIT2, IFI27, DDX58, EIF2AK2, IFITM1, RTP4, IFITM3, TRIM22, PARP12, IFIH1, OAS1, HERC6, RSAD2, DDX60, and MX2) were identified through bioinformatics and network analysis and are closely related to interferon production and function. Interestingly, immune cell infiltration analysis and correlation analysis demonstrate a positive correlation between the expression of 23 hub genes and monocyte infiltration in glomeruli and M2 macrophage infiltration in the tubulointerstitium of LN patients. Additionally, the CTD database, DsigDB database, and DREIMT database were used to explore the bridging role of genes in chemicals and LN as well as the potential influence of these chemicals on immune cells. After comparison and discussion, six small molecules (Acetohexamide, Suloctidil, Terfenadine, Prochlorperazine, Mefloquine, and Triprolidine) were selected for their potential ability in treating lupus nephritis.

## 1. Introduction

The pathogenesis of systemic lupus erythematosus (SLE) and lupus nephritis (LN) is generally caused by multiple factors including genetics, immune abnormalities, ultraviolet radiation, drugs, estrogen [1, 2], and viral infections [3, 4]. LN is characterized by glomeruli and tubulointerstitium inflammation [5], which are mediated by a variety of immune cells and cytokines [6]. This leads to a series of clinical presentations including hematuria, proteinuria, and impaired glomerular filtration rate [7].

At present, immunosuppressants, glucocorticoids (GC), and biological agents are mainly available for the treatment of LN worldwide. Various drug regimens have also been proposed based on the stage of the disease [8]. Since 1980, GC, mycophenolate (MPA), mofetil (MMF), calcineurin inhibitors (CNI), hydroxychloroquine (HCQ), rituximab (RTX), and others have been gradually explored in clinical practice and achieved promising results [9]. However, emerging studies are identifying their high side effect profile and toxicity. Some of them even fail to prevent disease recurrence in more than half of the patients [10]. Therefore, the discovery of new drugs for LN is of great urgency and importance to reduce mortality rates.

Currently, drug selection is primarily based on three principles: immunosuppression, immunomodulation, and symptomatic treatment [11]. Meanwhile, more studies are identifying genes that are implicated in the development and progression of LN [12], and researchers have sequenced kidney tissue from LN and documented them in the GEO database. Drug-gene relationships are continuously being enriched by further research and exploration.

In the present study, we focus on existing small molecules. First, we identify the core pathogenic genes of LN and then detect the association between chemicals, genes and LN through various reliable databases to screen potential small molecules for the treatment of LN. Finally, the potential mechanism of these small molecules was evaluated according to the immune signatures.

## 2. Materials and Methods

### 2.1. Microarray Data and Identification of Differently Expressed Genes (DEGs)

The screening criteria of datasets are as follows: First, the datasets must include cases and controls. Second, the organization used for sequencing should be the kidney of human. Third, the number of samples in each group should not be less than 10. Thus, GSE32591 [13] and GSE112943 [14] were downloaded from the GEO database [15]. Quality control was made to ensure our analysis accuracy. 46 glomeruli samples (32 disease samples and 14 control samples) and 47 tubulointerstitium samples (32 disease samples and 15 control samples) were obtained in the GSE32591 dataset. 21 kidney samples (14 disease samples and 7 control samples) were obtained in the GSE112943 dataset. We removed and/or averaged the probe sets which did not match the gene symbols or genes with multiple probe sets. Probes were transformed into the corresponding gene symbols under platform annotation information.

Differentially expressed genes between the LN and control groups in the GSE32591 and GSE112943 datasets were identified using the Limma R package [16]. Adjusted “*P* < 0.05 and Log (fold change) > 1 or Log (Fold Change) < −1” were defined as the thresholds for screening of the differential expression of mRNAs. The results of the two datasets are able to be combined into a more accurate target.

### 2.2. PPI Network and Identification of Hub Genes

The protein–protein interaction (PPI) network is an important means to identify protein functions and to understand system biology [17]. Search Tool for the Retrieval of Interacting Genes (STRING) (https://string-db.org/) was utilized to generate the PPI network of the common genes [18]. Analysis of functional interactions between proteins was performed to discover the mechanism of the occurrence and development of LN. Cytoscape (3.8.1) was developed for the visualization of molecular interaction networks [19] and better visualization of PPI network and identification of hub genes. Furthermore, we used the plug-in MCODE to perform PPIs network of hub genes and our selection criteria were as follows: MCODE scores > 5, node score cut‐off = 0.2, degree cut‐off = 2, *k*‐score = 2, and max depth = 100.

### 2.3. Gene Ontology and Kyoto Encyclopedia of Genes and Genomes Analysis

Visualization and Integrated Discovery (DAVID, http://david.ncifcrf.gov) [20] was employed to conduct Gene Ontology (GO), Kyoto Encyclopedia of Genes and Genomes (KEGG) pathway analysis to gain further insights into the biological pathways of the hub genes.

### 2.4. TF-miRNA Coregulatory Network

TF-miRNA coregulatory network analysis was performed to discover the potential expression mechanism of hub genes. Transcription factors (TFs) could act as both activators and repressors of gene expression at the transcriptional level, while miRNAs usually downregulated the expression of genes at the posttranscriptional level. TFs and miRNAs could regulate each other and coregulate a common target gene to form a forward loop (FFL) [21]. FFLs participated in many important cellular processes by regulating the expression of genes [22].

We used NetworkAnalyst (https://www.networkanalyst.ca/), a visual analytics platform for comprehensive gene expression profiling and meta-analysis [23], to identify TF-miRNA coregulatory interactions with identified hub genes. Interactions for TF-miRNA coregulation were collected from the RegNetwork repository, which is an integrated database of transcriptional and posttranscriptional regulatory networks in humans and mice [24]. Afterwards, TF-miRNA coregulatory network was visualized using Cytoscape.

### 2.5. Correlation Analysis between Hub Genes and Infiltrating Immune Cells

To further explore the correlation between hub genes and inflammation of the kidneys in LN patients, we uploaded the gene expression matrix data of GSE32591 to CIBERSORT. Immune cell infiltration analysis was performed on 46 glomeruli and 47 tubulointerstitium samples from the GSE32591 dataset and selected only samples with *P* < 0.05. R package “ggplot2” was used to draw the bar charts of 22 types of infiltrating immune cells and to visualize the differences in immune cell infiltration. Moreover, Spearman's correlation analysis on hub genes and infiltrating immune cells were performed using the OECloud tools at https://cloud.oebiotech.cn, and dot-chart was used to visualize the results.

### 2.6. Chemical-Gene-Disease Interactions

Understanding chemical-gene interactions could provide insight into the mechanisms of disease susceptibility of chemical actions and therapeutic drug interactions [25]. We obtained the interaction data between common chemicals, 23 hub genes, and LN from the Comparative Toxicogenomics Database (CTD, http://ctd.mdibl.org). CTD is a curated database that offers chemical-gene interactions, chemical-disease relationships, and gene-disease relationships from the literature for studying the effects of environmental chemicals on human health [26]. It is worth mentioning that chemicals in the CTD database include large and small molecules, as well as drugs and harmful substances. The figure was created with BioRender (https://biorender.com) [27] to help us visually understand and evaluate which common compounds may affect LN through identified hub genes. In addition, we used Cytoscape to create the network of small molecules and genes and calculate the degree of each gene and obtained the functions of 23 core genes from STRING.

### 2.7. Identification of Potential Small Molecules for LN

Identifying potential small molecules for LN is the ultimate target of our hub-gene screening. Drug Signatures database (DSigDB) which contains 22527 gene sets [28] was used to generate the small molecules which could downregulate the expression of hub genes. The access to the DSigDB database is acquired through Enrichr (https://amp.pharm.mssm.edu/Enrichr/) platform, an interactive and collaborative HTML5 gene list enrichment analysis tool [29].

Furthermore, DREIMT (http://www.dreimt.org.) is a bioinformatics tool for hypothesis generation and prioritization of drugs capable of modulating immune cell activity from transcriptomics data [30]. We used it to understand the immunological mechanisms by which the small molecules we predicted can affect LN patients. Meanwhile, we collected the immune signatures of seven drugs that have been shown to be effective in LN. Immune signatures can be widely used to better identify which small molecules are more reliable. BioRender was used to create the figure of immune signatures of potential small molecules.

## 3. Results

### 3.1. Identification of DEGs

**Figure 1:**
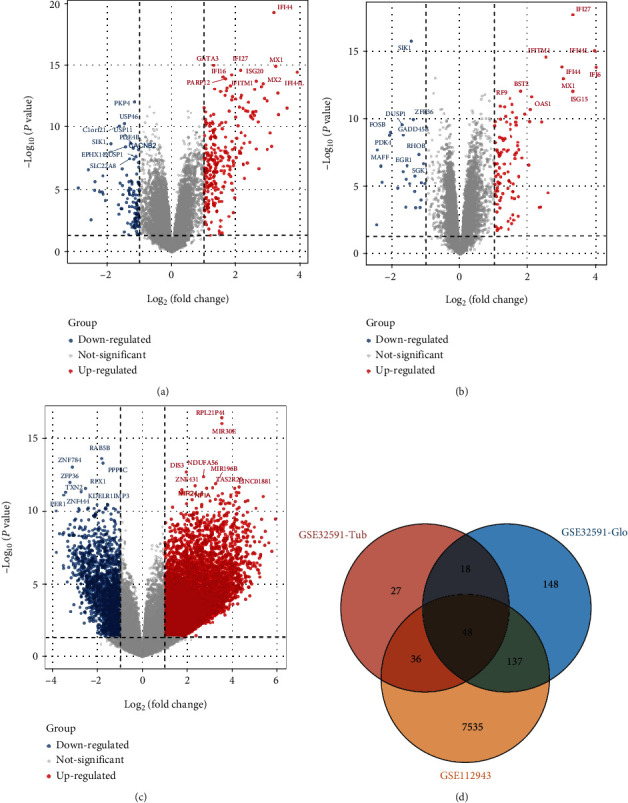
Identification of DEGs of kidney tissues from LN and control samples in GSE32591 and GSE112943. (a) The volcano map of all DEGs of the 46 glomeruli samples in GSE32591. (b) The volcano map of all DEGs of the 47 tubulointerstitium samples in GSE32591. (c) The volcano map of all DEGs of the 21 kidney samples in GSE112943. (d) The Venn diagram of GSE32591 and GSE112943.

Since three types of kidney tissues (Supplementary Table 1&2) are included in GSE32591 (glomeruli and tubulointerstitium) and GSE112943 (formalin-fixed paraffin-embedded kidney), we performed the DEGs of the three types of samples, respectively; 351 DEGs were identified in the glomeruli samples of GSE32591, with 250 upregulated genes and 101 downregulated genes (Figure 1(a)). Similarly, 129 DEGs were identified in the tubulointerstitial samples of GSE32591 (Figure 1(b)) of which 104 were upregulated and 25 were downregulated genes. Furthermore, a total of 7759 DEGs consisting of 6143 upregulated and 1616 downregulated genes were identified as significantly different in expression between the disease and control samples of GSE112943 (Figure 1(c)). Additionally, a total of 48 DEGs which comprises 45 common upregulated genes and 3 common downregulated genes were identified from the three types of samples (Figure 1(d)).

### 3.2. PPI Network and Identification of Hub Genes

**Figure 2:**
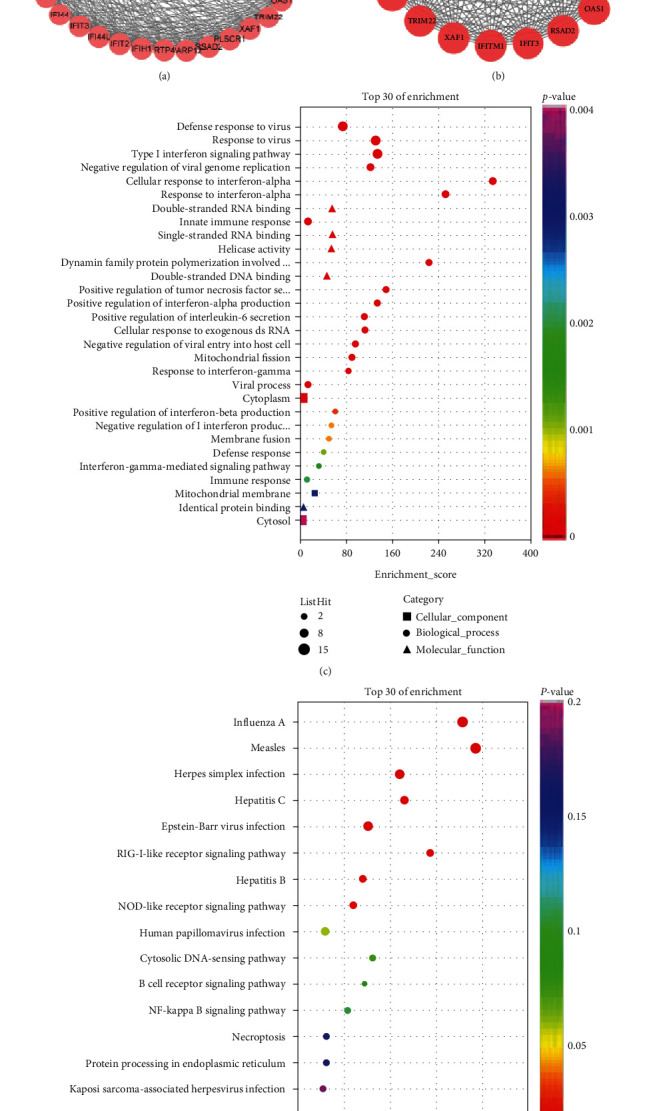
Protein–protein interaction (PPI) network and identification of hub genes. (a) Protein–protein interaction (PPI) network of 48 common genes in GSE32591 and GSE112943. Nodes in pink represent coupregulated genes while nodes in blue represent codownregulation of genes. The analyzed network holds 43 nodes and 296 edges. (b) Protein–protein interaction (PPI) network of 23 hub genes identified by MCODE in Cytoscape. The analyzed network holds 23 nodes and 240 edges. (c) Top 30 GO functional enrichment of the 23 hub genes. (c) 16 KEGG signaling pathway enrichment of the 23 hub genes.

The PPI network of common DEGs and most densely connected regions (43 nodes, 296 edges) were obtained from Cytoscape (Figure 2(a)). 23 genes (IFI6, PLSCR1, XAF1, IFI16, IFI44, MX1, IFI44L, IFIT3, IFIT2, IFI27, DDX58, EIF2AK2, IFITM1, RTP4, IFITM3, TRIM22, PARP12, IFIH1, OAS1, HERC6, RSAD2, DDX60, and MX2) were identified as hub genes using the plug-in MCODE in Cytoscape (Figure 2(b)). Since the products of genes were at the core of the PPI network, these hub genes were considered potential therapeutic targets.

### 3.3. Gene Ontology and Kyoto Encyclopedia of Genes and Genomes Analysis

To analyze the biological classification of DEGs, we performed a functional enrichment analysis of 23 hub genes. Functional enrichment analysis identified 101 GO terms in the biological process (BP) category and 33 GO terms in the cellular component (CC) category. Regarding BP, the hub genes were involved in viral defense response, type I interferon(IFN) signaling pathway, interferon-*α* (IFN-*ɑ*) response and positive regulation of tumor necrosis factor (TNF) secretion, and IFN-*α* production. In terms of MF, the DEGs were mainly associated with double-stranded RNA binding, single-stranded RNA binding, double-stranded DNA binding, and helicase activity. The cellular components of the DEGs were cytoplasm, mitochondrial membrane, cytosol, mitochondria, and mitochondrial outer membrane. The top 30 of GO enrichment were shown in Figure 2(c). Moreover, 16 KEGG pathway analysis indicates hub genes were mainly enriched in viral infections (Figure 2(d)).

### 3.4. TF-miRNA Coregulatory Network

**Figure 3:**
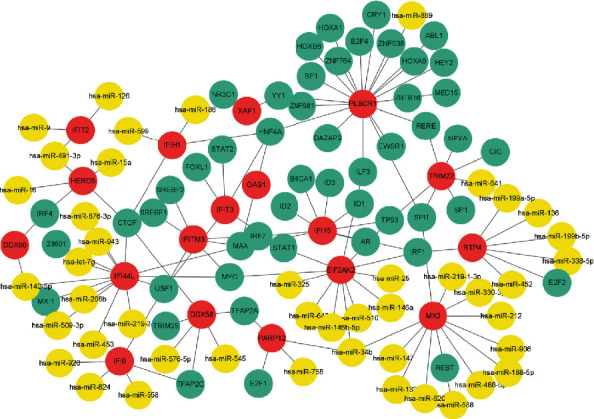
The network of TF-miRNA coregulatory. Nodes in red color represent the hub genes, nodes in blue color represent the TF-genes, and nodes in yellow color represent the miRNAs. The analyzed network holds 113 nodes and 127 edges. 55 miRNAs and 50 TF-genes have interacted with the 18 hub genes

A TF-miRNA coregulatory network was generated using NetworkAnalyst, and better visualization was seen through Cytoscape. The analysis of the TF-miRNA coregulatory network showed miRNA-TF interaction with the hub genes. The network created for TF-miRNA coregulatory network was performed in Figure 3, which contains 113 nodes and 127 edges. 45 miRNAs and 50 TF-genes have interacted with 18 hub genes. This network could provide us with reasonable regulatory mechanisms for the expression of the DEGs.

### 3.5. Correlation Analysis between Hub Genes and Infiltrating Immune Cells

**Figure 4:**
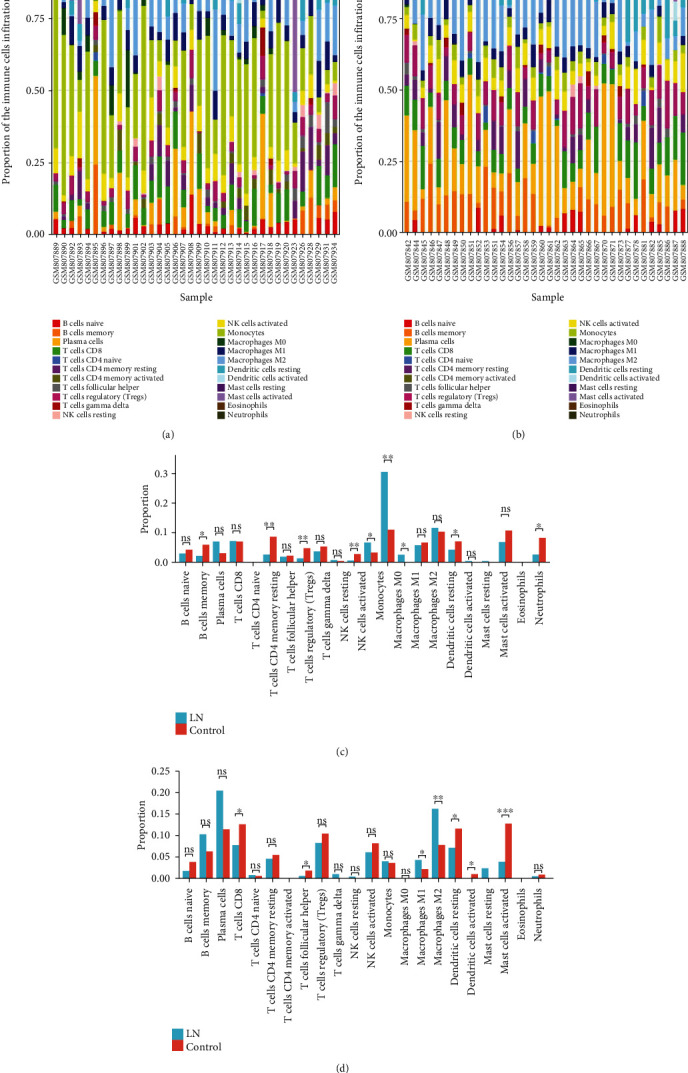
The landscape of immune infiltration in LN. (a) Bar charts of 22 immune cell proportions in LN and normal glomeruli tissues. (b) Bar charts of 22 immune cell proportions in LN and normal tubulointerstitium tissues. (c) Differential expression of different types of immune cells between LN and normal glomeruli tissues. (d) Differential expression of different types of immune cells between LN and normal tubulointerstitium tissues.

**Figure 5:**
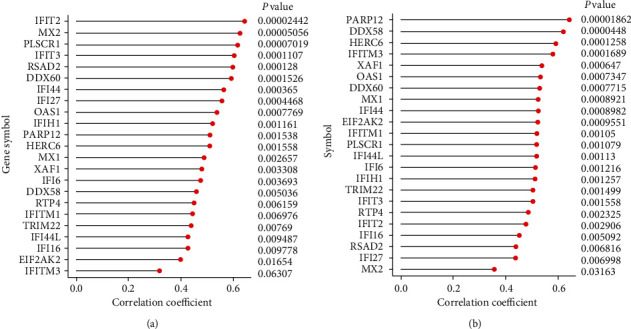
Correlation analysis between hub genes and infiltrating immune cells in glomeruli tissues (a) and tubulointerstitium tissues (b) of GSE32591.

Thirty-six glomeruli samples (30 disease samples and 6 control samples) and 36 tubulointerstitium samples (28 disease samples and 8 control samples) were maintained with *P* value < 0. 05. Figures 4(a) and 4(b) show the proportions of 22 immune cells in 36 glomeruli tissues and 36 tubulointerstitium tissues. Monocyte infiltration was predominant in the glomeruli (Figure 4(c)) with a statistically significant difference (*P* = 0.0065). Although the most infiltrated cell in tubulointerstitium was plasma cells, there was no statistically significant difference. Moreover, there was an abundant infiltration of M2 macrophages cells in the tubulointerstitium (*P* = 0.0042) (Figure 4(d)). Correlation analysis demonstrated a positive correlation between 23 hub genes and monocyte infiltration in the glomeruli and M2 macrophage infiltration in the tubulointerstitium (Figures 5(a) and 5(b)). The gene expression of LN was converted to infiltrating immune cells through CIBERSORT and after verification; the expression of 23 hub genes screened was positively correlated with the proportion of the most typical infiltrated immune cells in the glomeruli and tubulointerstitium of LN. This indicates that the 23 hub genes not only are representatives of the characteristic genetic effect but also signify the immune characteristics of LN.

### 3.6. Chemical-Gene-Disease Interactions

**Figure 6:**
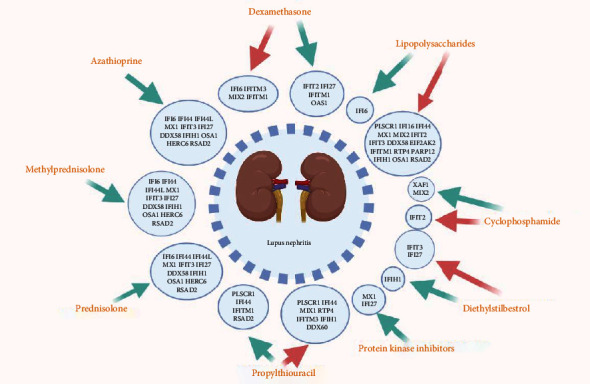
Chemical-gene-disease interactions. Nine common chemicals were identified through the CTD database that could affect LN by regulating the expression of 23 hub genes. The red arrows represent upregulated gene expression while the green arrows represent downregulated gene expression.

**Figure 7:**
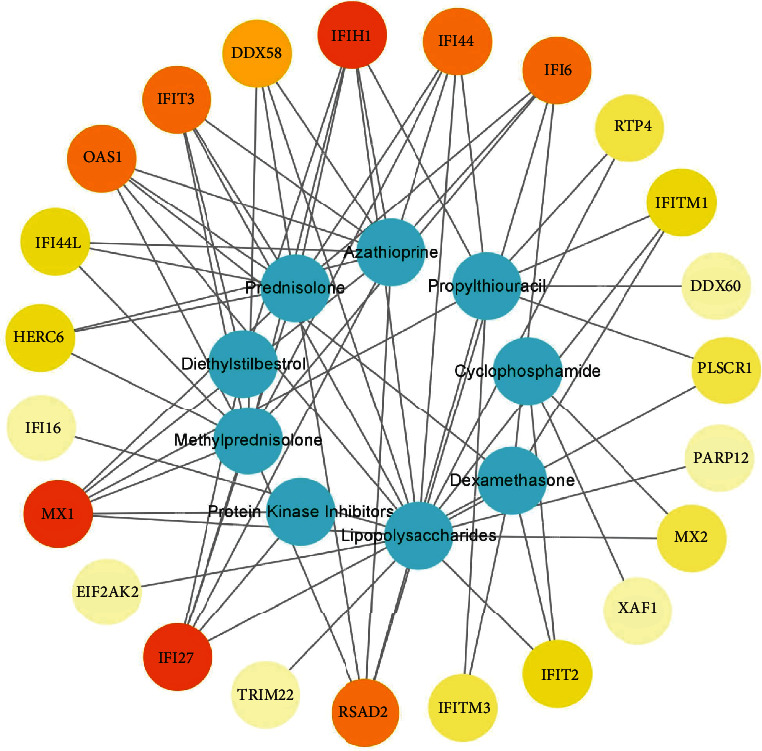
The network between chemicals and genes. Blue for chemicals; red for genes; the color depth of genes represents the level of degree.

Nine chemicals that affect LN by regulating the expression of 23 hub genes were identified through the CTD database (Supplementary Table 3), and their interactions are shown in Figure 6. Azathioprine, Lipopolysaccharides, Dexamethasone, Methylprednisolone, Cyclophosphamide, Prednisolone, Propylthiouracil, Diethylstilbestrol, and Protein Kinase Inhibitors were associated with LN by affecting the expression of hub genes. Among them, Azathioprine, Dexamethasone, Methylprednisolone, Cyclophosphamide, and Prednisolone could relieve LN by downregulating hub gene expression. It is noteworthy that the effects of Propylthiouracil on hub gene expression might be as complex as their duality. The data recorded in this database are mainly derived from experiments, and many of them consist of classical and common chemicals; many of the potential relationships are still being explored. Therefore, some chemicals closely related to LN may be unavailable, but we should not ignore their influence on the expression of hub genes. In addition, the network between chemicals and genes is shown in Figure 7. The color depth of genes represents the level of degree and their degree and function are available in Supplementary Table 4.

### 3.7. Identification of Potential Small Molecules

Enrichr platform is used to identify potential molecules for 23 hub DEGs. The small molecules which could downregulate the expression of hub genes were collected from the DSigDB database (Supplementary Table 5). The results from the potential small molecules were generated based on the odds ratio, which is automatically generated by the DSigDB database and represents the closeness between the small molecules and genes.  Table 1 points out the top 10 potential small molecules from the DSigDB database for hub genes.

**Figure 8:**
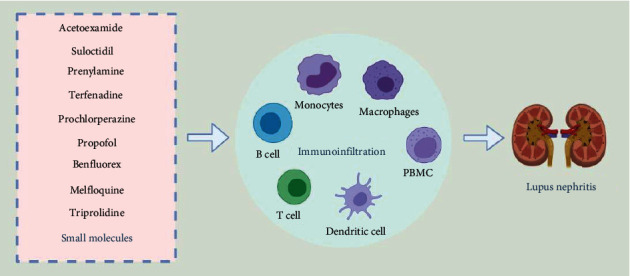
Immune signatures of potential small molecules. Nine small molecules may impact LN by altering immune cell infiltration including monocytes, macrophages, B cells, T cells, dendritic cells, and PBMC.

The DREIMT database provided an abundance of data between the relationship of potential small molecules and various immune cells (Supplementary Table 6). Nine of ten potential small molecules and 7 common effective drugs were found in the DREIMT database, and they mainly affect monocytes, macrophages, T cells, B cells, dendritic cells, and PBMC, which may be involved in the potential mechanism for the treatment of LN (Figure 8). The inhibition of immune cells by 9 potential and 7 common effective drugs is shown in Table 2.

## 4. Discussion

Approximately 70% of SLE patients have clinical manifestations of renal damage while 100% were found to have renal involvement when immunofluorescence and electron microscopy were performed on renal biopsy [41]. LN represents the most common complication of SLE, and renal involvement is significant in the prognosis of LN patients [42]. Accordingly, effective prevention and treatment of LN are of great importance and urgency.

23 common differential genes in tissue samples were identified in both datasets, making our results significant. The pathways of GO enrichment mainly involve the IFN signaling pathway, IFN-ɑ cellular response, innate immune response, and positive regulation of TNF and IFN-ɑ production. IFN is a primary pathogenic factor of LN [43] while TNF is a major player in the development of LN by inducing renal IgG deposition [44]. In addition, KEGG enrichment analysis showed that hub genes are enriched in viral infections such as influenza A, Epstein-Barr virus, and hepatitis B. These results suggest that the immune response to LN is similar to the human response towards viral infections. The results of the enrichment analysis demonstrated that drug prediction using 23 hub genes is reliable.

There are 50 TF-genes and 45 miRNAs in the TF-miRNA coregulatory network. Among the most interacted TFs, USF1, MAX, HNF4A, and CTCF have 4 edges. USF1 is associated with macrophage inflammation [45], and HNF4A is a major regulator of the renal proximal tubule [46]. Additionally, hsa-mir-34b, with 3 edges, is the most frequently reported epigenetically abnormal miRNAs in SLE [47]. Furthermore, researches have shown that FFL could affect the development of certain diseases, including cancer [48], by altering biological processes such as cell differentiation and cytokine production [49]. FFLs may affect the activity of IFN-related pathways in SLE through altered the expression of hub genes, which revealed the possibility of FFLs as novel biomarkers and therapeutic targets in LN.

The immune infiltration results suggest that the glomeruli of LN patients are dominated by monocyte infiltration in addition to a large amount of macrophage M2 cell infiltration in the tubulointerstitium, which was positively correlated with the expression of 23 hub genes. Monocytes are major players in both innate and adaptive immunities [50]. Their role in the inflammatory response is closely associated with glomerulopathy of LN [51] while macrophage M2 cells could promote kidney fibrosis [52]. It is worth noting that infections may increase monocytes [53] and that the widespread use of immunosuppression and steroid therapy in LN makes patients more susceptible to infections [11]. In addition, recent discoveries have demonstrated the importance of tubulointerstitial inflammation in LN. Both M1 and M2 macrophages are involved in the inflammation of tubulointerstitium. M1 macrophages are increased in LN compared to controls, and their histotoxicity leads to tubulointerstitial damage. Also, podocytes, mesangial cells, tubular epithelial cells, kidney resident macrophages, and stromal cells cause the produce cytokines and chemokines together which lead to their injury and damage of the kidney.

It is worth mentioning that the 23 hub genes obtained from three types of kidney tissue samples represent not only the genetic effect but also the emblematic immune signature of LN. The above results demonstrate they may be biomarkers and novel drug targets for the diagnosis and treatment of LN.

The result of chemical-gene-disease interactions demonstrated that nine chemicals could affect LN by regulating the expression of hub genes. Azathioprine, Cyclophosphamide, Dexamethasone, Methylprednisolone, and Prednisolone downregulate hub gene expression to treat LN. They are widely used in clinical therapy for LN and are proven safe and efficacious [54]. Moreover, Protein Kinase Inhibitors downregulate the expression of several hub genes, which could play an important anti-inflammatory role in autoimmune diseases [55].

In addition, natural diethylstilbestrol is a key player that not only affects the reproductive system but also markedly influences the immune system [56]. Estrogen is implicated in the pathogenic pathways in LN [57], and diethylstilbestrol may cause or worsen LN through the upregulation of hub genes, necessitating attention to the adverse effects of diethylstilbestrol. There has also been a report of LN occurring after treatment with Propylthiouracil suggesting that attention should be paid to Propylthiouracil in the clinical treatment of LN patients with hyperthyroidism.

Microbial studies have shown that many autoimmune diseases are infectious and lipopolysaccharides play a key role in host-pathogen interactions with the natural immune system [58]. Altered immune function induced by lipopolysaccharide could lead to enhanced immune responses in the kidney leading to renal insufficiency [59]. Lipopolysaccharide-gene-LN interactions may provide new mechanisms for the prevention and treatment of LN. Many chemicals are closely associated with LN, such as tacrolimus and hydroxychloroquine [60], and a few have been documented in the CTD database. It is important to note and explore their influence on the expression of hub genes in LN.

The mechanism of chemicals in the occurrence and development of diseases has long been a mystery, but genes may be a bridge between them. The function of 23 hub genes is closely related to the synthesis, secretion, and biological function of IFN. The presence of IFN-*α* in the serum of SLE patients can induce differentiation of normal monocytes into dendritic cells (DCS). This can capture apoptotic cells and nucleosomes and perform antigen presentation ultimately leading to the destruction of immune tolerance in LN. The occurrence of autoimmunity and a feedback loop of interaction centered around antigen-presenting cell-IFN-nuclear antigen plays an important link in the pathogenesis of SLE [43]. Chemicals may affect this pathway by regulating the expression of hub genes and ultimately contribute to LN. Azathioprine, Cyclophosphamide, Dexamethasone, Methylprednisolone, and Prednisolone are expected to downregulate the expression of hub genes to improve the abnormal immune state mediated by IFN. Other chemicals such as LPS may adversely affect LN by upregulating the expression of hub genes. Genes may be the language of conversation between chemicals and disease, and we should use them to predict potentially therapeutic small molecules for LN.

Small molecules which could downregulate the expression of 23 hub genes were found from the DSigDB database. Among all candidate small molecules, the current study highlights the top 10 key players: Acetohexamide, Suloctidil, Prenylamine, Terfenadine, Chlorophyllin, Prochlorperazine, Propofol, Benfluorex, Mefloquine, and Triprolidine.

Abnormal activation of immune cells is the most important feature of LN. Continuous activation of antigen-presenting cells (APC), imbalances of regulatory and effector CD4+T cells, and high proliferation and activity of B cells which secrete a lot of antibodies combined with autoantigen ultimately lead to autoimmunity of LN [61]. The immune signature of seven drugs commonly used to treat LN is due to their inhibition of B cell activation as well as helper T cells (Ths), regulatory T cells (Tregs), memory T cells (Tms), and APCs. Among them, hydroxychloroquine has inhibitory effects on plasma cells, memory B cells (Bms), naive B cells (Bns), NK cells, cytotoxic T cells (CTLs), Ths, Tms, naive T cells (Tns), APCs, and PBMCs, which may contribute to its central role in treating LN. A series of interactions between immune cells culminates in an increase in antibody secretion by B cells. Therefore, B cells play an indispensable role in LN, corresponding to the inhibition of B cells by common drugs. The nine small molecules we identified also show inhibitory effects on the relevant immune cells. Nevertheless, Prenylamine and Benfluorex are less effective due to their inability to inhibit B cell activity. Triprolidine displays an immune signature similar to that of HCQ which can inhibit T cell proliferation [62] demonstrating essential roles in the pathogenesis of LN. Other small molecules also show inhibition of relevant immune cells which signifies potential evidence for treating LN.

SLE is a systemic disease and the involvement of the nervous and circulatory systems may also change when LN occurs. Meanwhile, the occurrence of LN is accompanied by changes in blood glucose, lipids, and other physiological indicators [63]. Nevertheless, the current drugs for LN are mainly immunosuppressants, which can achieve straightforward effects, but can also cause many complications such as bone myelosuppression and liver damage. Therefore, there is a need to identify small molecules which may improve other symptoms of LN with fewer adverse effects. Acetohexamide could be effective in treating LN patients with diabetes on account of its hypoglycemic effects [31, 38]. Additionally, suloctidil may play a significant role in improving circulation and blood vessel function to benefit LN. Moreover, Propofol and Prochlorperazine may demonstrate unique effects in patients with Neurolupus, but Propofol is unlikely to be used, for it is a type of anesthetic. Also, the superior antioxidant and anti-mutational effects of chlorophyllin [35] may protect against kidney damage in LN. It is worth mentioning that mefloquine may have great therapeutic potential for LN as its analogue hydroxychloroquine plays an important role in the treatment of LN.

In conclusion, six small molecules (Acetohexamide, Suloctidil, Terfenadine, Prochlorperazine, Mefloquine, and Triprolidine) were considered meaningful to be validated in future trials as our current results have shown their rich potential in treating LN.

The GEO database provides little clinical information on the types of pathogenesis and disease activity of LN which is a limitation of our study. Further detailed information could make our conclusions more precise.

## 5. Conclusions

We identified six small molecules (Acetohexamide, Suloctidil, Terfenadine, Prochlorperazine, Mefloquine, and Triprolidine) that might have potential therapeutic effects for LN through the exploration of hub genes and immune characteristics of LN. The six small molecules can affect the immune signatures of LN by downregulating hub genes because 23 hub genes (IFI6, PLSCR1, XAF1, IFI16, IFI44, MX1, IFI44L, IFIT3, IFIT2, IFI27, DDX58, EIF2AK2, IFITM1, RTP4, IFITM3, TRIM22, PARP12, IFIH1, OAS1, HERC6, RSAD2, DDX60, and MX2) could emerge as the biomarkers and novel drug targets for the diagnosis and treatment of LN.

## Figures and Tables

**Table 1:** Suggested top 10 small molecules for the Lupus nephritis.

Small molecules	Odds ratio	*P* value	Category
Acetohexamide	2595.71	9.91*E* − 35	K-ATP inhibitors [31]
Suloctidil	1737.4875	3.40*E* − 44	Calcium channel blockers [32]
Prenylamine	1626.684783	3.01*E* − 37	Calcium channel blockers [33]
Terfenadine	673.9951691	7.41*E* − 31	H1 receptor blockers [34]
Chlorophyllin	293.4264706	6.63*E* − 13	Antioxidant [35]
Prochlorperazine	261.4460641	5.99*E* − 18	Dopamine receptor antagonists [36]
Propofol	231.084058	7.80*E* − 16	GABA receptor enhancer [37]
Benfluorex	230.9375	1.47*E* − 10	RNA polymerase II activator [38]
Mefloquine	168.0168421	3.10*E* − 08	Antimalarial [39]
Triprolidine	157.5631579	2.02*E* − 06	H1 receptor blockers [40]

**Table 2:** Immune signatures of 9 potential small molecules and 7 common effective drugs.

	Small molecules	Inhibited cells
	Acetohexamide	Bn, DC, macrophage, monocyte, PBMC, Pre-B2 cell, Th, Tn
	Suloctidil	Bm, Bn, DC, PBMC, Th1, Tm, Tn, Treg
	Prenylamine	DC, macrophage, CTL, PBMC, Th1, Tm, Treg
Potential small molecules	Terfenadine	Bn, DC, Th1, Th2, Tm, Treg
	Prochlorperazine	B1 cell, Bm, Bn, DC, macrophage, PBMC, Th1, Th2, Tm, Treg
	Propofol	Bn, DC, monocyte, PBMC, Tm
	Benfluorex	Macrophage, PBMC, Tn, Tm
	Mefloquine	Bm, Bn, DC, PBMC, Th1, Treg
	Triprolidine	B1 cell, Bn, DC, macrophage, PBMC, Pre-B1 cell, CTL, Th1, Th17, Tm, Treg
		
	Methylprednisolone	B cell, Pre-B2 cell, DC, macrophage, monocyte, neutrophil, NK cell, Tm, Tn, Treg
	Cyclophosphamide	Bm, DC, macrophage, monocyte, NK cell, PBMC, Th1, Th17, Tm, Tn
Common Drugs	Mycophenolate-mofetil	B cell, DC, neutrophil, NK cell, PBMC, Treg, Th1, Th2, Tm
	Azathioprine	B cell, DC, macrophage, monocyte, neutrophil, PBMC, Th1, Th2, Tm, Treg
	Hydroxychloroquine	Plasma cell, CD21B cell, Bms, Bn, macrophage, monocyte, neutrophil, NK cell, PBMC, CTL, Th, Tm, Tn
	Tacrolimus	Bm, Pre-B1 cell, Pre-B2 cell, DC, macrophage, monocyte, PBMC, CTL, Th1, Th2, Tm, Treg
	Cyclosporin-a	Bm, B1 cell, Pre-B2cell, DC, macrophage, monocyte, neutrophil, PBMC, CTL, Th1, Th2

Bn: naive B cell; Bm: memory B cell; Pre-B cell: precursor B cell; Tn: naive T cell: Th: helper T cell; Tm: memory T cell; Treg: regulatory T cell; CTL: cytotoxic T lymphocyte; DC: dendritic cell; PBMC: peripheral blood mononuclear cell; NK cell: natural killer cell.

## Data Availability

The raw data supporting the conclusions of this article will be made available in GEO database. All data generated or analyzed during this study are included in this article and its supplementary information files.

## Supplementary Materials

Supplementary Materials Supplementary Materials Supplementary Table1: the expression data of GSE32591, which contains 32 glomeruli and tubulointerstitum tissues of LN patients and 14 glomeruli tissues and 15 tubulointerstitum tissues of healthy controls. Supplementary Table2: the expression data of GSE112943 consists of formalin fixed paraffin-embedded kidneys (14 lupus nephritis and 7 control kidney). Supplementary Table3: the data of the relationships between Nine chemicals, 23 hub genes and lupus nephritis. Supplementary Table4: the degree of 23 hub genes in the network between small molecules and genes and the description and function of 23 hub genes. Supplementary Table5: all the data of small molecules based on the 23 hub genes from the DSigDB database. Supplementary Table6: the relationship of nine potential small molecules and 7 common effective drugs for LN were found in the DREIMT database.
